# Palindromic Vectors, Symmetropy and Symmentropy as Symmetry Descriptors of Binary Data

**DOI:** 10.3390/e24010082

**Published:** 2022-01-03

**Authors:** Jean-Marc Girault, Sébastien Ménigot

**Affiliations:** 1 Groupe ESEO, 49000 Angers, France; sebastien.menigot@eseo.fr; 2 Laboratoire d’Acoustique de l’Université du Mans (LAUM), UMR 6613, Institut d’Acoustique-Graduate School (IA-GS), CNRS, Le Mans Université, 72085 Le Mans, France

**Keywords:** palindrome, palindromic vectors, symmetry, symmentropy, symmetropy, entropy, complexity, descriptor, binary sequence

## Abstract

Today, the palindromic analysis of biological sequences, based exclusively on the study of “mirror” symmetry properties, is almost unavoidable. However, other types of symmetry, such as those present in friezes, could allow us to analyze binary sequences from another point of view. New tools, such as symmetropy and symmentropy, based on new types of palindromes allow us to discriminate binarized 1/f noise sequences better than Lempel–Ziv complexity. These new palindromes with new types of symmetry also allow for better discrimination of binarized DNA sequences. A relative error of 6% of symmetropy is obtained from the HUMHBB and YEAST1 DNA sequences. A factor of 4 between the slopes obtained from the linear fits of the local symmentropies for the two DNA sequences shows the discriminative capacity of the local symmentropy. Moreover, it is highlighted that a certain number of these new palindromes of sizes greater than 30 bits are more discriminating than those of smaller sizes assimilated to those from an independent and identically distributed random variable.

## 1. Introduction

The palindromic analysis of discrete sequences has partly revolutionized molecular biology and is widely used as shown by the following work [1,2,3,4,5,6,7,8], to name a few. Very recently, the study of quantum behavior [9], encountered in palindromes within the DNA structure, revealed that the symmetry properties of the unitary structure, other than those present in classical palindromes, play an important role in the origin and cause of mutations.

In the continuity of the work carried out by Tibatan and Sarisaman [9], our article aims to highlight the symmetry links between the concept of frieze and the concept of palindrome, which have been insufficiently exploited until now in the analysis of binary data.

The “mirror” symmetry on which the concept of palindrome was based is certainly the basis of the oldest symmetry descriptors. Its greatest success is undoubtedly derived from the analysis of biological sequences (DNA, RNA and proteins), even if in this case the definition of DNA palindromes is slightly different from the classical definition. (Let us consider the sequence of characters ‘ATGGCCAT’. It is qualified as a 8-palindrome sequence. It is composed of a 4-pattern on the right (‘CCAT’) obtained by a mirror reflection of its 4-pattern complementary on the left (‘ATGG’): ‘ATGG’|‘CCAT’, where $•|\underline{•}$ indicates the mirror reflection and $\underline{•}$ indicates the complementary. Note that ‘*T*’ is the complementary of ‘*A*’, and ‘*C*’ is the complementary of ‘*G*’).

To fix ideas, a palindrome of size *m*, called “*m*-palindrome”, is a discrete sequence composed of two contiguous symmetrical (mirror) sub-sequences each composed of *k*-patterns with k=⌊m/2⌋. For example, the alphabetic character sequence ddddddddbbbbbbb is a 16—palindrome composed of two 8—patterns: dddddddd,bbbbbbbb. Even if the theoretical research around the palindrome is still going on, as shown by the recent article by Gabric and Shallit [10] to name but a few, it is the older work of Allouche et al. [11,12], which is used as a starting point in this work and in particular the notion of **palindromic complexity**.

Today, when studying a word or a discrete sequence, its analysis is still limited to only one type of symmetry: the “mirror” symmetry. Wanting to extract many more intrinsic features in the discrete sequences studied can consist of looking for other types of symmetries, as it is explicitly the case in friezes.

A frieze is a horizontal strip composed of an infinite number of symmetrical patterns, i.e., a periodic geometric object. As an illustration, five types of alphabetical sequences of 16 characters, having the same symmetries as friezes, are presented as follows: bbbbbbbbbbbbbbbb, dbdbdbdbdbdbdbdb, bpbpbpbpbpbpbpbp, bqbqbqbqbqbqbqbq, bqpdbqpdbqpdbqpd.

If the objective is indeed to extend the analysis of discrete periodic sequences to other types of sequences, then the search for all symmetric patterns is the next step. To reach this goal, the concept of palindrome and then that of frieze is presented in Section 2. Then, the concept of palindromes is extended and new tools such as symmetropy and symmentropy are proposed in Section 3. Finally, the set of symmetry descriptors are tested on binarized 1/f noises and binarized DNA sequences in Section 4; then, the results are discussed in Section 5.

## 2. Palindromes and Friezes

In this section, we recall the concept of palindromes [11,12,13] and the concept of friezes [14,15].

### 2.1. Palindromes

For a binary sequence, an *m*-palindrome is, by definition, a grouping of *m* bits that form an *m*-pattern of mirror symmetry. In other words, for a binary sequence X={x(1),x(2),…,x(M)} composed of *M* bits, an *m*-palindrome can be defined as the concatenation of two *k*-patterns: $X_{m}\left(i\right)=[X_{k}\left(i\right)\;\Gamma_{R}\left[X_{k}\left(i\right)\right]$, with k=⌊m/2⌋ being the order of the palindrome. The first *k*-pattern $X_{k}\left(i\right)=\left\{x\left(i\right),x\left(i+1\right),\ldots ,x\left(i+k-1\right)\right\},1\leq i\leq M-k+1$ is the reference pattern, and the second *k*-pattern obtained by $\Gamma_{R}\left[X_{k}\left(i\right)\right]$ is the symmetric pattern, where $\Gamma_{R}\left[•\right]$ is the transformation corresponding to the mirror symmetry, a reflection.

For example, for the binary sequence X={01100110} of 8 bits, the first 4-pattern of X of order 2 is written as $X_{4}\left(1\right)=[X_{2}\left(1\right)\;\Gamma_{R}\left[X_{2}\left(1\right)\right]=\left[\left\{01\right\}\left\{10\right\}\right]=\left\{0110\right\}$, with $X_{2}\left(1\right)=\left\{01\right\}$ and $\Gamma_{R}\left[X_{2}\left(1\right)\right]=\left\{10\right\}$. In the same way, the 8-palindrome of X of order 4 is written as $X_{8}\left(1\right)=[X_{4}\left(1\right)\;\Gamma_{R}\left[X_{4}\left(1\right)\right]=\left[\left\{0110\right\}\left\{0110\right\}\right]=\left\{0110010\right\}$, with $X_{4}\left(1\right)=\left\{0110\right\}$ and $\Gamma_{R}\left[X_{4}\left(1\right)\right]=\left\{0110\right\}$.

A palindrome of odd length can be seen as the concatenation of a pattern of size (m−1) and its mirror, for which the rightmost bit of the (m−1)-reference pattern (bit in bold in the following example) and the leftmost bit of the (m−1)-mirror pattern (bit in bold in the following example) are merged to give only one. Example: [{01}{10}]={01 10} becomes {010}.

Although there is a plethora of scalar descriptors such as those indicated in [11,12,13] to name but a few, here, we limit ourselves to the concept of palindromic complexity $\tilde{c}$ computed from D, which lists, from the palindromic dictionary, the cardinal of the **different** palindromic words of size *m*:

(1) $$D={\left[d\left(0\right),d\left(1\right),\cdots ,d\left(m\right),\cdots ,d\left(M\right)\right]}^{t}.$$

where d(m) is the cardinal of “palindrome words” of size *m* [11] present in the binary sequence. The empty palindrome obtained for m=0 is *e* and {e,0,1} are the trivial palindromes. The **palindromic complexity**$\tilde{c}$, which corresponds to the cardinal of D, is defined by the following:

(2) $$\tilde{c}=card\left(D\right).$$

In order to measure the level of mirror symmetry present in a binary sequence, we propose to count the frequency of occurrence of *m*-palindromic patterns in the binary sequence studied by the following:

(3) $$V={\left[v\left(0\right),v\left(1\right),\cdots ,v\left(m\right),\cdots ,v\left(M\right)\right]}^{t}.$$

where v(m) is the frequency of occurrence of a palindromic pattern of size *m*. The “mirror” symmetry level $\tilde{\sigma }$ is the sum of all occurrences for non-trivial palindromes:

(4) $$\tilde{\sigma }=\sum_{m=2}^{M}v\left(m\right).$$

An illustration given in Table 1 for the binary sequence X={01101001}, specifies the value of the palindromic complexity $\tilde{c}=5$. There are, in all, five non-zero elements in D={1,2,2,2,2,0,0,0,0} which is itself computed from the empirical palindromic dictionary Dict={e,0,1,00,11,010,101,0110,1001}.

### 2.2. Friezes

**Figure 1:**
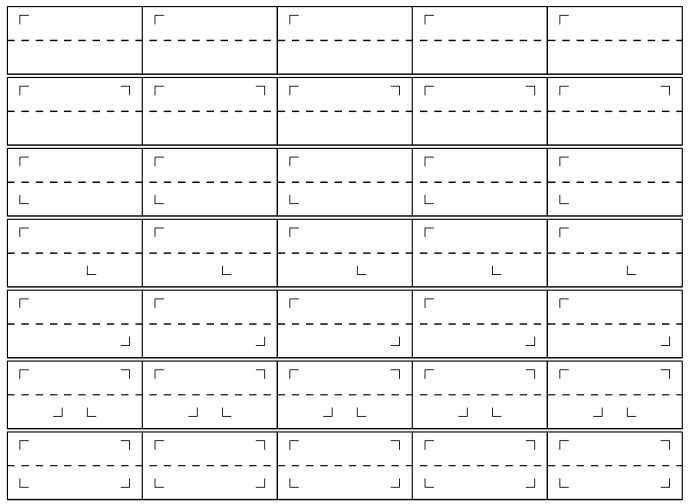
The seven types of friezes with a ⌜ pattern. The friezes 1, 2, 4, 5 and 6 can constitute periodic discrete sequences because no pattern appears with the same abscissa. This is not the case for friezes 3 and 7, which cannot constitute a discrete sequence. Among the five periodic sequences, friezes 2 and 6 are composed of palindromes.

As stated in the Introduction, a frieze is a periodic horizontal band composed of a few basic symmetrical patterns repeated *ad infinitum*. There are only seven different types of friezes [14,15] (see Figure 1) obtained from five types of isometries (isometry is a geometrical transformation that leaves the objects invariant thus transformed while preserving the distances, which is the case for the five following operations: translation, vertical reflection, horizontal reflection, inversion, and glide reflection). (**TRIGH**: **T**ranslation, vertical **R**eflection, **I**nversion and **G**lide reflection, **H**orizontal reflection). There are only 5 possible types of periodic discrete sequences obtained from 4 types of isometries (**TRIG**: **T**ranslation, vertical **R**eflection, **I**nversion and **G**lide reflection), vertical reflection not allowing to obtain a 1D-sequences.

For example, from the friezes in Figure 1 and replacing ⌜ by {10}, we can construct five types of periodic discrete sequences, all having different types of symmetry:

- sequence X={10101010⋯} obtained with translations;
- sequence X={10011001⋯} obtained with vertical reflections (mirror);
- sequence X={10011001⋯} obtained with glide reflections and translations;
- sequence X={10101010⋯} obtained with inversions and translations;
- sequence X={10100101⋯} obtained with inversions and vertical reflections.

Among the five previous sequences, two are composed of mirror palindromes (the second and the last). By no longer limiting the search to mirror palindromes, it should be possible to describe binary sequences more precisely; this is the subject of the next section.

## 3. Methods

In this section, we propose to extend the different palindromic vector and scalar descriptors by integrating the different types of symmetry revealed in the friezes. Then, new palindromic descriptors such as the notions of symmetropy and symmentropy are proposed.

As mentioned later, through the notion of friezes, several types of symmetries can be considered using the combination of only four isometries (TRIG). On this basis, we propose to generalize the notion of palindromes by taking into account all types of symmetries.

For a binary sequence X={x(1),x(2),…,x(M)} composed of *M* bits, an *m*-palindrome of type j∈{T,R,I,G} can be defined as the concatenation of two *k*-patterns: $X_{m}\left(i\right)=\left[X_{k}\left(i\right)\;\Gamma_{j}\left[X_{k}\left(i\right)\right]\right]$ with k=m/2. The first *k*-pattern $X_{k}\left(i\right)=\left\{x\left(i\right),x\left(i+1\right),\ldots ,x\left(i+k-1\right)\right\},1\leq i\leq M-k+1$ is the reference pattern, and the second *k*-pattern is the one obtained by one of the four isometries $\Gamma_{j}\left[X_{k}\left(i\right)\right]$ with j∈{T,R,I,G}:

- $\Gamma_{T}\left[X_{k}\left(i\right)\right]]=\left\{x\left(i\right),x\left(i+1\right),\ldots ,x\left(i+k-1\right)\right\}$. A translation is simply a “copy and paste”;
- $\Gamma_{R}\left[X_{k}\left(i\right)\right]]=\left\{x\left(i+k-1\right),\ldots ,x\left(i+1\right),x\left(i\right)\right\}$. A vertical reflection is simply a “copy, return and paste”;
- $\Gamma_{I}\left[X_{k}\left(i\right)\right]]=\underline{\left\{x(i+k-1),\ldots ,x(i+1),x(i)\right\}}$. An inversion is simply a “copy-complement-return-paste”;
- $\Gamma_{G}\left[X_{k}\left(i\right)\right]]=\underline{\left\{x(i),x(i+1),\ldots ,x(i+k-1)\right\}}$. A glide reflection is simply a “copy-complement-paste”;

where $\underline{•}$ is the logical function NOT, also called a complement. For example, with the binary sequence X={01010101}, the first 4-palindrome of type ‘*T*’ is written as $X_{4}\left(1\right)=\left\{0101\right\}$, with $X_{2}\left(1\right)=\left\{01\right\}$ and $\Gamma_{T}\left[X_{2}\left(1\right)\right]=\Gamma_{I}\left[X_{2}\left(1\right)\right]=\left\{01\right\}$.

If the objective is to measure the level of symmetry of a binary sequence through the presence of palindromes of type j∈{T,R,I,G}, then we can define the following measure:

(5) $$v_{j}^{*}\left(m\right)=\frac{v_{j}\left(m\right)}{2(M-m+1)(M-1)}$$

with $v_{j}\left(m\right)$ being the total number of palindromes of size *m*, $v_{j}\left(0\right)=M$ and the palindrome vector of type *j* by the following:

(6) $$V_{j}^{*}={\left[v_{j}^{*}\left(0\right),v_{j}^{*}\left(1\right),\cdots ,v_{j}^{*}\left(m\right),\cdots ,v_{j}^{*}\left(M\right)\right]}^{t}.$$

In order to propose a scalar measure of the level of symmetry of a given type, it seems judicious not to take into account the non-trivial palindromes because they could mask, for very long sequences, the presence of larger palindromes in smaller numbers. The total number of non-trivial palindromes $\sigma_{j}^{*}$ of type j∈{T,R,I,G}, for the whole range of sizes *m*, is obtained by computing

(7) $$\sigma_{j}^{*}=\sum_{m=2}^{M}v_{j}^{*}\left(m\right).$$

To obtain the global level of symmetry present in a binary sequence, the global palindromic symmetropy $\sigma^{*}$ is defined as follows:

(8) $$\sigma^{*}=\sigma_{T}^{*}+\sigma_{R}^{*}+\sigma_{I}^{*}+\sigma_{G}^{*},$$

where $\sigma_{R}^{*}=\tilde{\sigma }$ is defined in Section 2. Note that, for binary sequences where the level of symmetry is the maximum as for example for the sequences X={01010101} and X={111111}, the symmetropy is maximum with $\sigma^{*}=1$.

To quantify the “diversity” of different types of palindromes, the overall palindromic **symmentropy** E can be defined as follows:

(9) $$E=-P^{t}\log_{4}P,$$

where P is the *quarte* probability P defined as follows:

(10) $$P={\left[p_{T},p_{R},p_{I},p_{G}\right]}^{t},$$

with $p_{j}=\sigma_{j}/\sigma^{*}$. Note that the values of the symmentropy are between 1/2 and 1. When there is equi-probability, then E=1. For example, for the sequence X={01010101} of M=8 bits, the symmentropy is maximal at E=0.99, and the value of $E=\lim_{M\to \infty }1$. When two probabilities out of four are null with $P={\left[1/2,1/2,0,0\right]}^{t}$, as is the case for the 8-bit sequence X={11111111}, then the symmentropy is minimal and is E=1/2. This means that, when the symmentropy is minimal, there is always a minimum symmetric information content in the binary sequences.

Finally, it seems appropriate to compute a local palindromic symmentropy ϵ(m) for each *m* scale:

(11) $$\epsilon \left(m\right)=-Q^{t}\left(m\right)log_{4}Q\left(m\right),$$

where $Q\left(m\right)={\left[q_{T}\left(m\right),q_{R}\left(m\right),q_{I}\left(m\right),q_{G}\left(m\right)\right]}^{t}$ is the *quarte* probability at scale *m*, where $q_{j}\left(m\right)=\frac{v_{j}\left(m\right)}{\sigma (m)}$ and with $\sigma \left(m\right)=v_{T}\left(m\right)+v_{R}\left(m\right)+v_{I}\left(m\right)+v_{G}\left(m\right)$.

To illustrate, let us consider the binary sequence X={01101001} of 8 bits. We reported in Table 2 $Dict_{j}$, $v_{j}\left(m\right)$, $v_{j}^{*}\left(m\right)$ and $q_{j}$ with j∈{T,R,I,G}.

Remark: This measure of symmentropy is similar in idea to the one proposed by Yodogawa [16], who proposed an entropic measure of the level of symmetry present in the images via a decomposition in the Walsh–Hadamard basis. (The method of Yodogawa that measures the entropy of symmetric patterns is called symmetropy. From our point of view, it is rather a symmentropy since it is derived from an entropy measure, which is not the case of symmetropy as we define it in Section 3. On the other hand, in Yodogawa’s approach, the probabilities allowing us to computation the entropy in base 2 are obtained from a decomposition in the Walsh–Hadamard basis. In Yodogawa’s paper, it is clearly stated that not all symmetries are considered, which is not the case for our approach based on symmetry friezes.) Here, the proposed definition is different.

## 4. Results

In this section, we wish to show the interest of these new scalar and vector descriptors in the study of binarized sequences. We propose to compute the different proposed descriptors (palindromic vectors, symmetropy and symmentropy) for binarized sequences taken from 1/f noises and 2 DNA sequences.

### 4.1. Binarized 1/f Noise

One way to study complexity, in which the meaning here is reduced to that of irregularity as reported in [17], is to vary the exponent β of the noise in $f^{\beta }$. For β=0, the generated noise is white noise, and for β=−2, the generated noise is a Brownian motion, with the integral of a white noise being a Brownian motion.

Here, in order to stay within the framework of our study, the time series are binarized. All values above the median are replaced by ‘1’, otherwise ‘0’. Moreover, in order to compare the different scalar and vector descriptors, the Lempel–Ziv complexity $C_{lz}$ is proposed as a reference and is computed as presented in [18]. This normalized complexity is almost zero for periodic binary sequences and close to unity for random sequences such as white noise.

**Figure 2:**
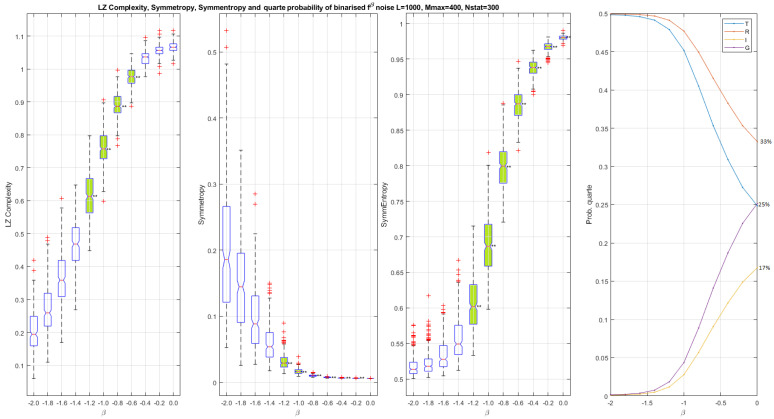
Scalar palindromic descriptors obtained from binarized $f^{\beta }$ noises and for different values of β. Left, Lempel–Ziv complexity $C_{lz}$, in which the boxes do not overlap for −1.2<β<−0.6. Left middle, symmetropy $\sigma^{*}$, in which the boxes do not overlap for −1.2<β<−0.4. Right middle, symmentropy E, in which boxes do not overlap for β>−1.2. Left, *quarte* probability P versus β. When β=−2, the *quarte* probability is P=[0.50,0.50,0.00,0.00]. When β=0, the *quarte* probability is P=[0.25,0.33,0.17,0.25] and the level of reflection symmetry is higher than the translation/ glide reflection and the inversion: $\sigma_{R}^{*}>\sigma_{T}^{*}=\sigma_{G}^{*}>\sigma_{I}^{*}$. The closer β is to zero, the higher the complexity. We notice that both $C_{lz}$ and E increase as the complexity increases. On the contrary $\sigma^{*}$ decreases as the complexity increases.

**Figure 3:**
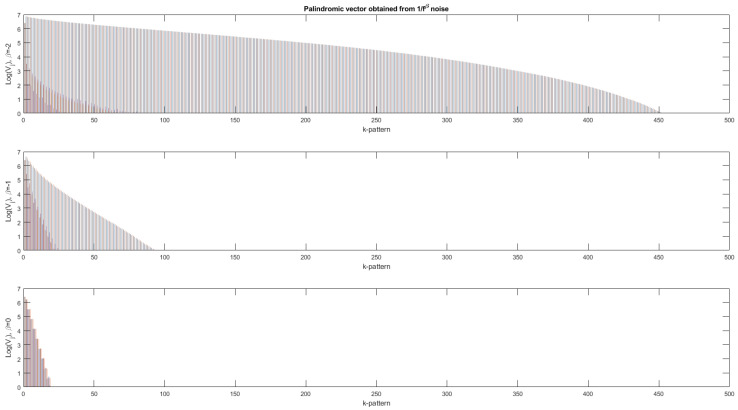
Average palindromic vectors obtained for binarized $f^{\beta }$ noises of length 1000, with β=−2.0,−1.0,−0.0 (from top to bottom) and m∈{1:500}. Top, average palindromic vectors obtained after averaging 300 vectors for β=−2.0. Middle, average palindromic vectors obtained after averaging 300 vectors for β=−1.0. Bottom, average palindrome vectors obtained after averaging 300 vectors for β=0.0. The more irregular the sequence (strong negative value of β) and the larger the spread of the palindromic vector descriptors.

**Figure 4:**
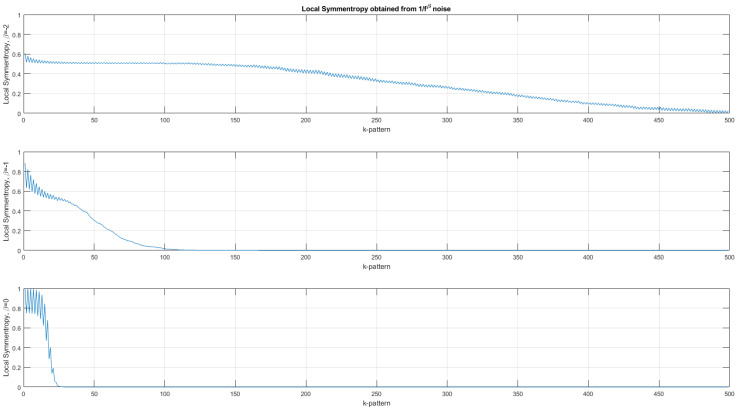
Average local symmentropy (with 300 trials) computed for three types of noises. Top, local symmentropy of a Brownian motion (β=−2). Middle, local symmentropy of a pink noise (β=−1). Bottom, local symmentropy of a white noise (β=0). The sawtooth fluctuation comes from the fact that the symmentropy values are slightly different for even and odd palindromes. The local symmentropy synthesizes the information carried by the four palindromic vectors into only one.

In Figure 2, Figure 3 and Figure 4, the scalar and vector descriptors obtained for noises in $f^{\beta }$ with β∈{−2:0} by step of 0.2 are presented. For a same value of β, 300 binarized noises composed of 1000 bits are generated.

In Figure 2, the different scalar palindromic descriptors are computed and plotted as whisker boxes. From Figure 2, we observe that all scalar palindromic descriptors describe monotonic curves increasing for Lempel–Ziv complexity and symmentropy and decreasing for symmetropy (as well as these components through the *quarte* probability P). This monotonicity property can be auspicious for tracing the values of β knowing the value of the descriptor. Indeed, it is possible to discriminate binarized noises in $f^{\beta }$ on larger or smaller regions depending on the descriptor considered. For example, for the Lempel–Ziv complexity, the body (second and third quartile) of the non-overlapping whisker boxes in the region −1.2<β<−0.6 allows us, from a Lempel–Ziv complexity of 0.62, to go back to a value of β=−1.2 without much error. When β=0, the complexity is maximal and tends to unity; when β=−2, the complexity is less and is 0.2 for a Brownian motion. For symmetropy, the non-overlapping boxes for −1.2<β<−0.4 also allows us to find the value of β from the symmetropy measures. Note that the discrimination range (β>−1.2) of symmentropy is much larger than those obtained by Lempel–Ziv complexity and symmetropy. We also check that the values of the symmentropy are well between 1/2 and 1. Finally in Figure 2, we observe a decrease in the probabilities drawn from the *quarte* P. Indeed, it decreases as β approaches zero for types ‘T’ and ‘R’ to go from 50% to 25% and 33%, respectively, and it increases progressively for types ‘I’ and ‘G’ to go from 0% to 17% and 25%, respectively. At the maximum complexity β=0, we observe that the reflection symmetry level is always higher than the translation/glide reflection and inversion: $\sigma_{R}^{*}>\sigma_{T}^{*}=\sigma_{G}^{*}>\sigma_{I}^{*}$.

**Figure 5:**
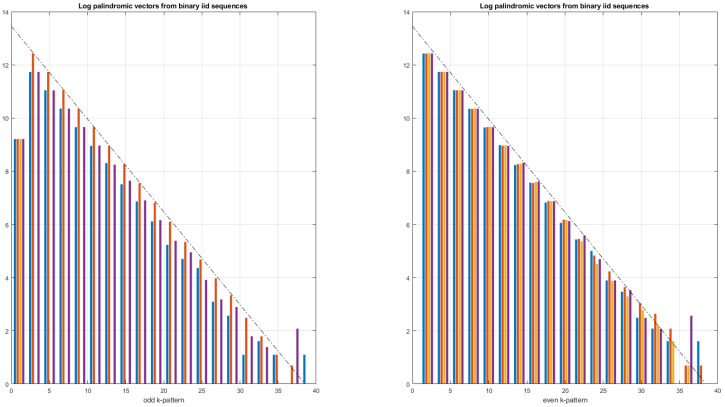
Logarithm of the four average palindromic vectors computed from 100 binary sequences iid (independent and identically distributed) of 5000 bits. We note a different distribution between even and odd palindromes. There is an equi-distribution between the different types of symmetry for even palindromes. For odd palindromes, we also note the non-presence of palindromes of ‘I’ type. We note a decrease in the symmetry levels as the size of the palindromes increases. Note that there are no palindromes with sizes exceeding 40. Finally, on average, the proportion of palindromes is $P_{T}=25\%,P_{R}=33\%,P_{I}=17\%$ and $P_{G}=25\%$.

In Figure 3, the palindromic vectors obtained for β=−2,−1,0, which correspond to Brownian motion, pink noise and white noise, respectively, are presented. From Figure 3, we observe that all of the average palindromic vectors (obtained by averaging 300 palindromic vectors) decrease as the palindromic size *m* increases and this decrease is all the more marked as β approaches zero, i.e., when the correlations between samples are almost non-existent. Note that, for Brownian motions (β=−2), there are large palindromes up to about 450. On the contrary, for white noise, we note that the size of the palindromes does not exceed 20 bits. Moreover, the palindromic vector obtained for β=0 is very similar to the one obtained in the case of binary iid (independent and identically distributed) sequences, as shown in Figure 5.

In Figure 4, the local symmentropy (averaged from 300 trials) ϵ(m) computed for three different types of noise (Brownian motion, pink noise and white noise) is plotted. As for the palindromic vectors, the symmentropy decreases as the size of the palindromes increases. The spread out of the symmentropy depends on the type of noise and thus on the correlations between samples. The range in size is very small for white noise with no correlation between samples/bits compared with Brownian motion. Moreover, the value of the symmetropy is close to unity for the white noise and close to half for the Brownian motion.

In Figure 5, the palindromic vectors obtained from binary sequences independent and identically distributed are plotted. We observe in Figure 5 a different distribution between even and odd palindromes. There is an equi-distribution between the different types of symmetry for the even palindromes. For odd palindromes, we also note the non-presence of palindromes of type ‘I’. Note that there are no palindromes with sizes exceeding 40 bits. On average, the proportion of palindromes is $P_{T}=25\%,P_{R}=33\%$, $P_{I}=17\%$, $P_{G}=25\%$. We notice a decrease in the symmetry levels as the size of the palindromes increases. In logarithmic scale, the decrease in the symmetry level (and thus of the number of symmetrical palindromes) is linear. Indeed, for a fixed length of the binary sequence, the more the palindrome size increases, the smaller the number of palindromes composing the binary sequence. For example, a sequence of 8 bits can only be composed of one palindrome of size m=8, of two palindromes of size m=4, of four palindromes of size m=2 and of eight palindromes of size m=1. This decrease is therefore inversely proportional to the size *m*. If we suppose that, for a given type of symmetry, the palindrome vector is expressed by $V_{j}\left(m\right)=K_{j}/m$, then $log\left(V_{j}\left(m\right)\right)=-1\times log\left(m\right)+log\left(K_{j}\right)$. This is indeed the affine line observed in Figure 5.

### 4.2. Biological Sequences: DNA

To show the relevance of the different symmetry descriptors proposed in a practical case, let us consider two DNA sequences. The objective is to identify descriptors that allow us to differentiate the two sequences: HUMHBB (human β-region, chromosome 11) with 73,308 bases and YEAST1 (Saccharomyces cerevisiae yeast, chromosome 1) with 230,209 bases obtained from (http://ncbi.nlm.nih.gov (accessed on 30 December 2021)). The DNA sequences is binarized, ‘A’ and ‘G’ are coded by 1, and ‘T’ and ‘C’ are coded by 0. For example, the sequence ‘ATATGCATTTCC⋯’ is coded ‘101010100000’.

At first, it seems interesting to indicate that, although the sequence “YEAST1” is 3.14 larger than the sequence “HUMHBB”, the total number of palindromes coming from the sequence “YEAST1” is 2.95 times larger than that of the sequence “HUMHBB”, as indicated in Table 3.

Moreover, we notice in Table 3 that the proportion of palindromes of type “mirror” (i.e., ‘R’ type) is much higher than that in the other types regardless of the DNA sequence considered. This corroborates what has been observed for 1/f noises, namely $P_{R}>P_{T}>P_{G}>p_{I}$, where $P_{j}$ is the palindromic probability of type *j*.

In Table 4, the Lempel–Ziv complexity $C_{lz}$, the symmentropy E and the symmetropy $\sigma^{*}$ are reported. From Table 4, we notice that the scalar descriptors are slightly different for the 2 DNA sequences. We note a relative difference of 4% for the Lempel–Ziv complexity (4%=(0.98−0.94)/0.94), of 1% for the symmentropy (1%=(0.97−0.96)/0.96) and of 6% for the symmetropy (6%=(0.85−0.80)/0.80)

**Figure 6:**
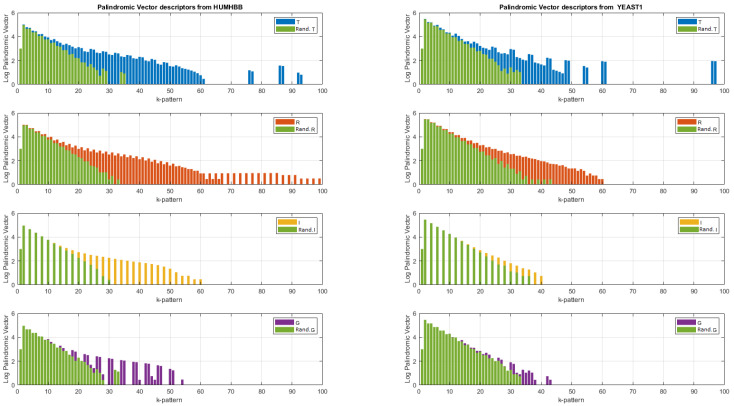
Logarithm of the palindromic vectors obtained from the entirety of the two DNA sequences for $m_{max}=500$. Zoom for m∈(1,100). In green, logarithm of the palindromic vectors obtained after randomization of the DNA sequences.

To go further in the analysis of DNA sequences, in Figure 6, the palindromic vector descriptors for each type *j* for m∈(0,100) are reported, even if the calculation has been made with $m_{max}=500$. We notice that the palindromic vectors are rather concentrated in the 0–100 band with some peaks (not shown here) beyond m=100 located in m=270,192 for “YEAST1” and m=124 for “HUMHBB”. As for the noises in 1/f, we notice a different distribution of the types of palindromes. For example, there are no more palindromes of type ‘R’ for the sequence “YEAST1” beyond m=60, idem for the palindromes of type ‘I’ for the sequence “YEAST1” beyond m=40. By the way, note that there are no even palindromes of type ‘I’. This shows the importance of taking into account all types of palindromes and not only the “mirror” palindromes of type ‘R’. By superimposing the palindromic vectors obtained after randomization, we can better see the “useful” information. The signature after randomization being similar to that of an independent and identically distributed random variable seems to be less important information and therefore useless for DNA sequence discrimination.

**Figure 7:**
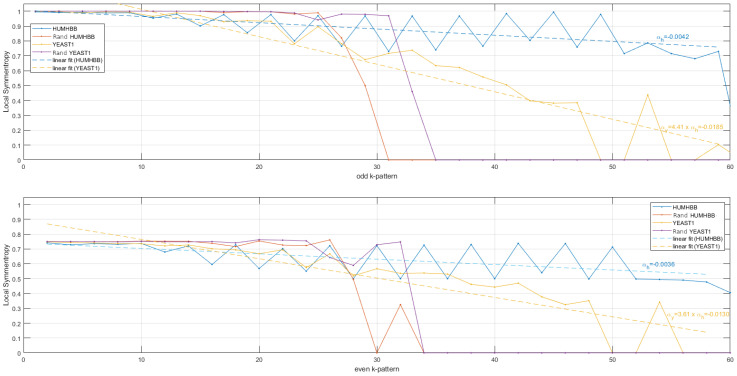
Local symmentropies obtained from binarized DNA sequences in the scale range m∈{1,60}. Top, odd palindromes. In blue, local symmentropy obtained from HUMHBB and straight line fitting. In orange, local symmentropy obtained from YEAST1 and straight line fitting. In magenta, local symmentropy obtained from randomized HUMHBB. In red, local symmentropy obtained from randomized Yeast. The slope $\alpha_{y}$ derived from the linear fitting for YEAST1 is 4.41 times the slope $\alpha_{h}$ obtained from HUMHBB. Bottom, even palindromes. The slope $\alpha_{y}$ derived from the linear fitting for YEAST1 is 3.61 times the slope $\alpha_{h}$ obtained from HUMHBB.

Finally, it seems interesting to show how local symmentropies allow us to differentiate each DNA sequence. In Figure 7, the local symmentropies calculated from “HUMHBB”, randomized “HUMHBB”, “YEAST1” and randomized “YEAST1” are reported. Straight lines derived from linear fitting from symmentropies show slopes that are significantly different between each DNA sequence. Indeed, from odd palindromes, the slope derived from the linear fitting for YEAST1 is 4.41 times the slope obtained from HUMHBB. For even palindromes, the slope derived from the linear fitting for YEAST1 is 3.61 times the slope obtained from HUMHBB. As expected, symmentropies obtained from randomized DNA sequences are similar while m<20 and close to unity. Indeed, the binary sequence obtained after randomization is very similar to an independent and identically distributed random variable for which the symmentropy is maximal and worth unity. For m>30, as shown in Figure 7, the symmentropies between the 2 DNA sequences are different.

## 5. Discussion and Conclusions

In this work, we proposed new palindromic descriptors (scalar and vector). The notions of palindromic vectors, palindromic symmetropy and palindromic symmentropy have been tested with binarized 1/f noises and 2 DNA sequences. For $f^{\beta }$ noises for which the “complexity” level is adjustable via β, we showed that palindromic symmetropy as well as palindromic symmentropy allows us to better discriminate the different $f^{\beta }$ noises on a larger range than the Lempel–Ziv complexity. Moreover, we showed that symmentropy is a complexity descriptor very similar to the Lempel–Ziv complexity. However, the palindromic symmetropy indicates the level of symmetry and is a descriptor of “anti-complexity”.

From this preliminary study, we notice that the “mirror” symmetry is more present than the other types of symmetries regardless of the level of complexity (see Figure 2). This is probably why only the “mirror” symmetry through the classical notion of palindrome has been considered so far. However, we showed (see Figure 6) that the four types of palindromes are necessary to better discriminate the binary sequences. Moreover, we showed that the distribution of the types of palindrome evolves with complexity. It goes from 50% for ‘T’ and ‘R’ types and 0% for ‘I’ and ‘G’ types when the complexity is low to 25%, 33%, 17% and 25% for ‘T’, ‘R’, ‘I’ and ‘G’ types when the complexity is maximal. These values are found when the binarized DNA sequences have been randomized.

Multiscale palindromic exploration, i.e., for the whole *m* size range of palindromes, through palindromic vectors and local symmentropy, allows us to go further in the analysis of binary sequences. In particular, it allows us to highlight a particular signature of independent and identically distributed random binary sequences found for white noise (β=0) and in the two DNA sequences. This exploration also allows us to clearly identify regions that allow us to discriminate the two DNA sequences. Furthermore, a factor of 4 between the slopes of the linear fits of the local symmentropies calculated from the two DNA sequences shows the discriminative capacity of the local symmentropy.

It seems obvious, as in the article by Tibatan and Sarisaman [9], that symmetry properties, insufficiently exploited to date, play a more important role in the exploration of biological sequences, both at the molecular and sub-molecular levels. The new palindromic descriptors presented in this work should contribute in a non-negligible way and should be widely applied in the study of biological sequences.

## Figures and Tables

**Table 1:** Dict, d(m) and v(m) calculated from the binary sequence X={01101001} composed of M=8 bits. There are in total $\tilde{c}=5$ sizes of palindromes (0,1,2,3,4) derived from the dictionary and used in the binary sequence X. There are two palindromes of size 2, two palindromes of size 3, and two palindromes of size 4, so a total of $\tilde{\sigma }=6=2+2+2$ palindromes composing the binary sequence.

*m*	0	1	2	3	4	5	6	7	8
Dict	*e*	0,1	00,11	101,010	0110,1001	-	-	-	-
d(m)	1	2	2	2	2	0	0	0	0
v(m)	8	8	2	2	2	0	0	0	0

**Table 2:** $Dict_{j}$, $v_{j}\left(m\right)$, $q_{j}$ and ϵ(m) with j∈{T,R,I,G} computed from the 8 binary sequence X={01101001}. The non-trivial palindromic symmetropy is $\sigma^{*}=0.44=1302/2940$ with $\sigma_{T}^{*}=102/2940$, $\sigma_{R}^{*}=214/2940$, $\sigma_{I}^{*}=472/2940$ and $\sigma_{G}^{*}=514/2940$, and the global palindromic symmentropy is $E=0.89=-\left(\frac{102}{1302}log_{4}\left(\frac{102}{1302}\right)+\frac{214}{1302}log_{4}\left(\frac{214}{1302}\right)+\frac{472}{1302}log_{4}\left(\frac{472}{1302}\right)+\frac{514}{1302}log_{4}\left(\frac{514}{1302}\right)\right)$.

*m*	0	1	2	3	4	5	6	7	8
$Dict_{T}$	e	0,1	00,11	-	1010	-	-	-	-
$v_{T}\left(m\right)$	8	8	2	0	1	0	0	0	0
$v_{T}^{*}\left(m\right)$	-	-	$\frac{2}{2\times 7\times 7}$	$\frac{0}{2\times 6\times 7}$	$\frac{1}{2\times 5\times 7}$	$\frac{0}{2\times 4\times 7}$	$\frac{0}{2\times 3\times 7}$	$\frac{0}{2\times 2\times 7}$	$\frac{0}{2\times 1\times 7}$
$q_{T}\left(m\right)$	-	-	2/14	0	1/6	-	0	-	0
$Dict_{R}$	*e*	0,1	00,11	101,010	0110,1001	-	-	-	-
$v_{R}\left(m\right)$	8	8	2	2	2	0	0	0	0
$v_{R}^{*}\left(m\right)$	-	-	$\frac{2}{2\times 7\times 7}$	$\frac{2}{2\times 6\times 7}$	$\frac{2}{2\times 5\times 7}$	$\frac{0}{2\times 4\times 7}$	$\frac{0}{2\times 3\times 7}$	$\frac{0}{2\times 2\times 7}$	$\frac{0}{2\times 1\times 7}$
$q_{R}\left(m\right)$	-	-	2/14	1/2	2/6	-	0	-	0
$Dict_{I}$	*e*	0,1	01,10	-	1010	-	110100	-	01101001
$v_{I}\left(m\right)$	8	8	5	0	1	0	1	0	1
$v_{I}^{*}\left(m\right)$	-	-	$\frac{5}{2\times 7\times 7}$	$\frac{0}{2\times 6\times 7}$	$\frac{1}{2\times 5\times 7}$	$\frac{0}{2\times 4\times 7}$	$\frac{1}{2\times 3\times 7}$	$\frac{0}{2\times 2\times 7}$	$\frac{1}{2\times 1\times 7}$
$q_{I}\left(m\right)$	-	-	5/14	0	1/6	-	1	0	1/2
$Dict_{G}$	*e*	0,1	01,10	010,101	0110,1001	-	-	-	01101001
$v_{G}\left(m\right)$	8	8	5	2	2	0	0	0	1
$v_{G}^{*}\left(m\right)$	-	-	$\frac{5}{2\times 7\times 7}$	$\frac{2}{2\times 6\times 7}$	$\frac{2}{2\times 5\times 7}$	$\frac{0}{2\times 4\times 7}$	$\frac{0}{2\times 3\times 7}$	$\frac{0}{2\times 2\times 7}$	$\frac{1}{2\times 1\times 7}$
$q_{G}\left(m\right)$	-	-	5/14	1/2	2/6	-	0	-	1/2
ϵ(m)	-	-	0.93	0.50	0.96	-	0	-	0.50

**Table 3:** Distribution in % of the total number of palindromes of different types present in each of the two non-randomized and randomized DNA sequences, m∈[1,500]. For the non-randomized sequences, the most frequent palindromes are reflection palindromes with $N_{R}>N_{T}>N_{G}>N_{I}$, while for the randomized sequences, the distribution is $N_{R}>N_{T}=N_{G}>N_{I}$. The distribution of the different types of palindromes is very similar regardless of the type of DNA sequence. The differences between the total number of palindromes from non-randomized and randomized HUMHBB and YEAST1 sequences are 496,028 − 441,299 = 54,729 and 1,463,633 − 1,384,396 = 79,237, respectively.

DNA seq	$N_{T}/N_{\mathrm{Total}}$	$N_{R}/N_{\mathrm{Total}}$	$N_{I}/N_{\mathrm{Total}}$	$N_{G}/N_{\mathrm{Total}}$	$N_{\mathrm{Total}}$
HUMHBB	29.5%	36.8%	13.5%	20.2%	496,028
randomized HUMHBB	24.9%	33.3%	16.7%	25.1%	441,299
Yeast1	27.9%	35.5%	14.5%	22.1%	1 463 633
randomized Yeast1	25.0%	33.3%	16.7%	25.0%	1,384,396

**Table 4:** Scalar palindromic descriptors of binarized DNA sequences. Lempel–Ziv complexity $C_{lz}$, symmentropy E and symmetropy $\sigma^{*}$ with m∈{0,500}. From scalar palindromic descriptors, it seems possible to differentiate the 2 DNA sequences. The values of Lempel–Ziv complexity and symmentropy are close to unity, indicating a high level of complexity. For randomized DNA sequences, Lempel–Ziv complexity and symmentropy tend toward unity.

DNA seq	$C_{\mathrm{lz}}$	E	$100\times \sigma^{*}$
HUMHHB	0.94	0.96	0.85
randomized HUMHHB	1.02	0.98	0.75
Yeast1	0.98	0.97	0.80
randomized Yeast1	1.01	0.98	0.75
